# Estimates of Dengue Force of Infection in Children in Colombo, Sri Lanka

**DOI:** 10.1371/journal.pntd.0002259

**Published:** 2013-06-06

**Authors:** Clarence C. Tam, Hasitha Tissera, Aravinda M. de Silva, Aruna Dharshan De Silva, Harold S. Margolis, Ananda Amarasinge

**Affiliations:** 1 Department of Epidemiology and Population Health, London School of Hygiene and Tropical Medicine, London, United Kingdom; 2 Epidemiology Unit, Ministry of Health, Colombo, Sri Lanka; 3 University of North Carolina School of Medicine, Chapel Hill, North Carolina, United States of America; 4 Genetech Research Institute, Colombo, Sri Lanka; 5 Pediatric Dengue Vaccine Initiative, International Vaccine Institute, Seoul, South Korea; 6 Centers for Disease Control and Prevention Dengue Branch, San Juan, Puerto Rico; University of California, Davis, United States of America

## Abstract

Dengue is the most important vector-borne viral disease worldwide and a major cause of childhood fever burden in Sri Lanka, which has experienced a number of large epidemics in the past decade. Despite this, data on the burden and transmission of dengue virus in the Indian Subcontinent are lacking. As part of a longitudinal fever surveillance study, we conducted a dengue seroprevalence survey among children aged <12 years in Colombo, Sri Lanka. We used a catalytic model to estimate the risk of primary infection among seronegative children. Over 50% of children had IgG antibodies to dengue virus and seroprevalence increased with age. The risk of primary infection was 14.1% per year (95% CI: 12.7%–15.6%), indicating that among initially seronegative children, approximately 1 in 7 experience their first infection within 12 months. There was weak evidence to suggest that the force of primary infection could be lower for children aged 6 years and above. We estimate that there are approximately 30 primary dengue infections among children <12 years in the community for every case notified to national surveillance, although this ratio is closer to 100∶1 among infants. Dengue represents a considerable infection burden among children in urban Sri Lanka, with levels of transmission comparable to those in the more established epidemics of Southeast Asia.

## Author Summary

Dengue is an increasing problem in the Asian subcontinent, but little research exists on dengue burden and transmission in this region. Dengue ranges from mild fever to pronounced circulatory shock and potentially death. However, clinical disease gives an incomplete picture of how much dengue is circulating, because many infections are asymptomatic. Presence of antibodies to dengue virus provides evidence of past infection. By studying how antibody prevalence changes with age, the force of infection can be estimated, a key measure of population transmission that quantifies the risk of a first infection among dengue-naive (seronegative) individuals. We estimated the force of dengue primary infection by applying a catalytic model to data from a serological study of children in Colombo, Sri Lanka. Over 70% of children experienced at least one infection by the age of 12 years, and the median age at infection was 4.7 years. Among dengue-naive children 14% can be expected to experience a dengue infection within 12 months. The high force of infection at young ages indicates a very high level of dengue virus transmission in this urban setting that is comparable with levels seen in other regions with well-established epidemics, including Southeast Asia and Latin America.

## Introduction

Dengue is considered to be the most important mosquito-borne viral disease affecting humans today [1]. Between 50–100 million cases occur worldwide each year, resulting in an estimated 500,000 hospitalizations and 20,000 deaths; approximately two-thirds of the world's population lives in areas colonized by *Aedes* mosquitos, the principal vector for dengue viruses [2].

Dengue viruses thrive in urban areas that support large *Aedes* populations and close contact between infectious vectors and susceptible human hosts [1], [3]. Dengue was first serologically confirmed in Sri Lanka in 1962, with the first island-wide outbreak being reported in 1965 [4]. Although Sri Lanka has had a history of over 40 years of dengue, since the early 2000s, progressively large epidemics have occurred at regular intervals. Dengue transmission in Sri Lanka is endemic, but unusually large epidemics were experienced in 2004 and 2009 with the peak transmission occurring in June, following the southwesterly monsoon. Dengue is now considered to be hyperendemic in Sri Lanka, involving co-circulation of multiple serotypes [5], [6]. In 2012, 44,456 dengue cases were notified, corresponding to a rate of 220 per 100,000 population; approximately a quarter of notified cases occur in children under 15 years. Despite this, little is known about the epidemiology of dengue and the transmission of dengue viruses among children in Sri Lanka, in whom the risk of severe forms of the disease, including dengue haemorrhagic fever (DHF) and dengue shock syndrome (DSS), is considerably higher. In this paper, we estimate the risk of dengue primary infection among dengue-naive individuals using data from a seroprevalence survey in the paediatric population of Colombo, Sri Lanka.

## Methods

### Ethics statement

Ethical approval for the study was obtained from the Ethical Review Committee of the Faculty of Medicine, University of Colombo. Permission to conduct the study was obtained from the Special Commissioner of the Colombo Municipality and the Chief Medical Officer of Health, Municipal Council Colombo. Ethical approval was also obtained from the following institutions: The Human Subjects Protection Committee of the Pediatric Dengue Vaccine Initiative, International Vaccine Institute, Korea; The Research Committees of the Lady Ridgeway Children's Hospital and Medical Research Institute, Sri Lanka; The Advisory Committee on Communicable Diseases, Ministry of Health, Sri Lanka; The Ethical Review Committee of the University of North Carolina, Chapel Hill, USA; The Ethical Review Committee of the London School of Hygiene & Tropical Medicine, UK. History of vaccination against Japanese Encephalitis (JE) virus was ascertained by parental consent and verified from vaccination records where available. Signed, informed consent to take part in the study was obtained from the child's parent or guardian.

### Seroprevalence survey

A census of Municipal Council Ward 33 (MCW33), Colombo, was carried out between September and October 2008 to enumerate all households and children <12 years in the area. MCW33 covers an area of 37.3 km^2^ and comprises a relatively homogeneous population of low-income families. The population of children <12 years at the last census in 2001 was 4737. Prior to the census, a digital map of the area was constructed and the area divided into census blocks. In each block, a designated field worker visited every residence and obtained information on all children <12 years living in the household and socioeconomic characteristics of the household.

Following the census, 802 children from 504 randomly selected households were recruited into the study. Selection of households within blocks was proportional to population size. Recruitment took place between November 2008 and January 2009. Children were excluded if they were at increased risk of harm following a blood draw, had hemophilia, leukemia or thrombocytopenic purpura. At enrolment, a baseline dengue seroprevalence survey was conducted. A fingerprick blood sample was taken from every child on filter paper strips for antibody testing (Dried Blood Spot Saver Cards manufactured by ID Biological Systems, Greenville, South Carolina, USA: Ref IDBS1003). Each sample was labeled, air dried and transported in a special container to the study laboratory at the end of the day. History of vaccination against Japanese Encephalitis (JE) virus was ascertained from parents or legal guardians and verified from vaccination records where available. The data used in this analysis come from this baseline seroprevalence survey.

### Laboratory analysis

The dried blood spot samples were stored with desiccants at −20°C until testing. Dengue virus seropositivity was determined by dengue IgG ELISA as previously described [7] and OD values ≥0.3 were considered flavivirus antibody positive.

### Data analysis

This analysis includes data from 797 children for whom age on the date of the baseline sample could be confirmed. We used a catalytic model to estimate the dengue force of infection among dengue naive children from age-specific seroprevalence data [8]. In the context of dengue, this approach has a specific interpretation. Dengue can result from infection with any one of four viral serotypes. Infection with one serotype provides long-term protection to that serotype, but not to others. Unlike for other common childhood immunizing infections, therefore dengue seropositive individuals could still be susceptible to secondary, heterotypic infections. However, ELISA tests cannot distinguish between dengue serotypes and current dengue diagnostics cannot reliably determine how many infections an individual has experienced. For the purposes of this analysis, we use the term “force of infection” specifically to mean the annual risk of infection with any serotype among dengue-naive (seronegative) individuals. This is equivalent to the rate of seroconversion, and we use the two terms interchangeably.

The catalytic model predicts that the proportion of seronegative individuals declines with increasing age at a constant rate, λ, according to the relationship:
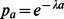
where *p_a_* is the proportion seronegative by age *a* years. Assuming that the risk of infection is constant over time, the force of infection parameter, λ, defines the rate at which seroconversion (first infection) increases with age. The above model can be expressed within a generalized linear modelling framework, such that:
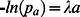
where λ can be estimated by maximum likelihood regression methods. The model can be generalized to allow the force of infection to vary with age. We fitted two different models to our seroprevalence data, Model 1 assuming a constant force of infection, and Model 2 the force of infection to vary with age:
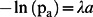
(Model 1)

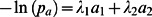
(Model 2)In Model 2, *a_1_* and *a_2_* are linear terms of age for two different age groups, 0.5 to <6 years and 6 to <12 years respectively; *λ_1_* and *λ_2_* are estimates of the force of infection in each age group. *a* and *a_1_* are constrained to be ≥0.5 years, as seropositivity below this age is likely to represent presence of maternal antibody. We estimated the force of infection parameters, with corresponding 95% confidence intervals (95% CIs) using logistic models with a log link. Although our survey sample included children from the same household, accounting for household level clustering made a negligible difference to our estimates. We therefore ignored the clustering, and used the likelihood ratio (LR) test to determine the level of statistical support for the model with age-varying force of infection over the model assuming a constant force of infection.

### Sensitivity analysis

We investigated the sensitivity of Model 2 to different age groupings by varying the age breakpoint by one year above and below the cut-off of 6 years. We used AIC to compare model fit, favouring the model with the lowest AIC value.

We also investigated the effect of JE vaccination history, to determine whether there was evidence that JE seropositivity interfered with the flavivirus ELISA. We hypothesized two possible scenarios: 1) that a substantial fraction of flavivirus positive ELISA results were due to infection with JE virus rather than dengue virus; 2) that the results of the flavivirus ELISA were influenced by the presence of vaccine-derived antibodies to JE virus. In either scenario, we expected that the force of infection estimates would be different between JE vaccinees and non-vaccinees, which would be manifested in a statistical interaction between JE vaccination history and age. Conversely, if the flavivirus ELISA results are not influenced by JE seropositivity, no such interaction should be present, and the force of infection estimates should be similar in both JE vaccinees and non-vaccinees. We evaluated the association between JE vaccination and flavivirus seropositivity using the χ^2^ test, and compared the median age of JE-vaccinated and non-vaccinated children using the Kruskall-Wallis test. We then fit logistic models as above, but additionally including terms for the interaction between age and JE vaccination status, to determine whether force of infection estimates differed between JE vaccinees and non-vaccinees. We compared the age-specific force of infection parameters for vaccinees and non-vaccinees, and used the LR test to assess evidence for the interaction term. We also fitted models including JE vaccination as a covariate, to assess potential confounding of the force of infection parameter by JE vaccination status. We compared the force of infection parameters from models with and without the JE vaccination variable to assess evidence of confounding, and used the Wald test to assess evidence of an effect of JE vaccination history on serological status.

### Ratio of dengue infections to notified cases

The number of dengue infections far exceeds the number of clinical and notified cases, because many infections are asymptomatic and only a fraction of clinical dengue cases seek medical care and are notified to the authorities. We estimated, for the Colombo Municipal Council (CMC), the ratio of infections to notified cases for the year 2009. We obtained the expected size of the paediatric population, by single year of age, by applying an annual population growth factor of 0.7% to population estimates from the 2001 census (Department of the Census, Sri Lanka, personal communication). To estimate the number of dengue primary infections, we applied our age-specific estimates of force of infection to the age distribution of the paediatric population in CMC, discounting from each single-year age group the number of seroconversions expected to have occurred in the previous year. We divided the age-specific number of primary infections by the number of DF/DHF cases notified to the Ministry of Health in 2009 to obtain the ratio of infections to notified cases. Because of difficulties in differentiating between infection and maternal antibody, we excluded infants <6 months old from the analysis.

## Results

Table 1 shows the number and percentage of children positive for flavivirus antibodies. Overall 51.4% (407) of children were seropositive. There was an increasing trend with age, from 7.4% (2) among infants between 6 and 12 months to 71.7% (38) by the age of 11 years. Among 25 infants <6 months of age, 33.3% were seropositive, indicating that there is substantial acquisition of maternal antibodies among infants in this population. The median age at seroconversion was 4.7 years.

**Table 1:** Number and percentage of children positive for flavivirus antibodies, Colombo, Sri Lanka, 2008–9.

				95% CI	
Age (years)	Seropositive	Total	% positive	Lower	Upper
**<0.5**	8	25	33.3%	15.6%	55.3%
**0.5**	2	28	7.4%	0.9%	24.3%
**1**	11	59	19.0%	9.9%	31.4%
**2**	17	59	28.8%	17.8%	42.1%
**3**	28	73	38.9%	27.6%	51.1%
**4**	22	57	38.6%	26.0%	52.4%
**5**	36	71	50.7%	38.6%	62.8%
**6**	38	67	56.7%	44.0%	68.8%
**7**	49	73	67.1%	55.1%	77.7%
**8**	60	91	65.9%	55.3%	75.5%
**9**	46	66	69.7%	57.1%	80.4%
**10**	52	74	70.3%	58.5%	80.3%
**11**	38	54	71.7%	57.7%	83.2%

### Logistic model

**Figure 1:**
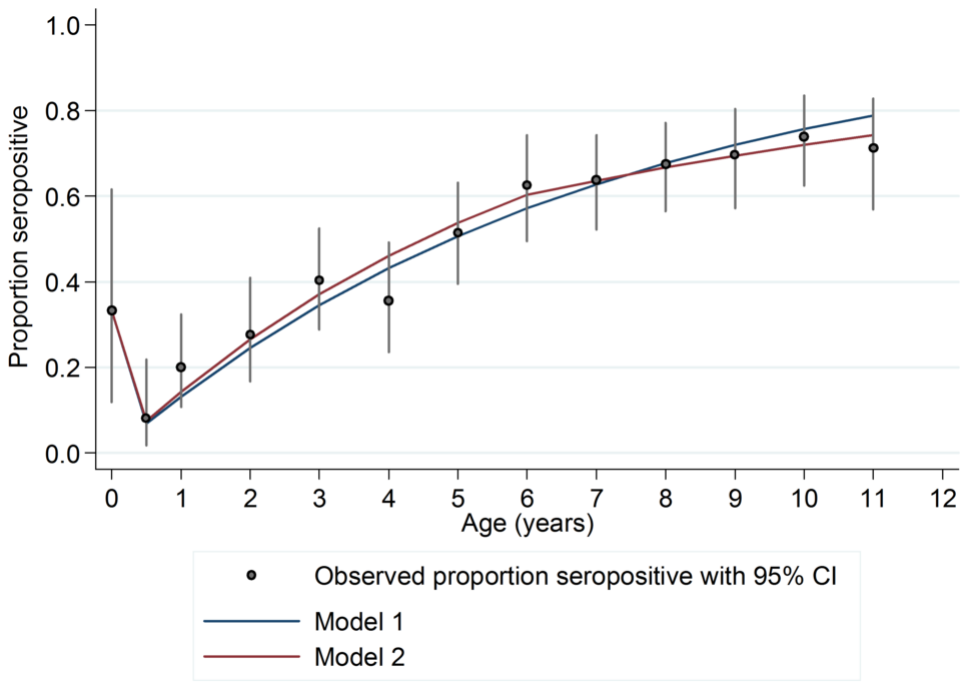
Observed prevalence by age (with 95% CIs) and model-predicted values. Blue line: Model 1 (constant force of infection); Red line: Model 2 (different forces of infection above and below 6 years).

Force of infection estimates from Models 1 and 2 are shown in Table 2. Assuming a constant force of infection (Model 1), dengue-naïve children seroconvert at a rate of 14.1% per year (95% CI: 12.7%–15.6%). Under Model 2, the annual rate of seroconversion was 15.4% (95% CI: 13.2%–17.7%) among children aged 6 months to <6 years, and 8.7% (95% CI: 2%–15.4%) among children aged 6 to <12 years. The LR test showed only weak evidence to favour Model 2 (p = 0.111). Similarly, a z-test comparing the *λ_1_* and *λ_2_* parameters from Model 2 showed only weak evidence that these were significantly different (p = 0.109). The model predictions are plotted together with the observed age-specific seroprevalences (and 95% CIs) in Figure 1. Despite weak evidence in to support Model 2, the predictions from this model appear to provide a slightly better fit to the observed seroprevalence values, particularly for children above 6 years.

**Table 2:** Force of infection estimates for Models 1 (constant force of infection) and 2 (age-varying force of infection).

				95% CI				LR test
Model	Parameter	Description	Estimate	Lower	Upper	p-value	AIC^1^	p-value^2^
Model 1	λ	Constant force of infection	0.141	0.127	0.156	<0.001	1.233	
Model 2	λ_1_	Force of infection between 6 months and <6 years	0.154	0.132	0.177	<0.001	1.232	0.111
	λ_2_	Force of infection between 6 and <12 years	0.087	0.020	0.154	0.011		

1 AIC: Akaike's Information Criterion;

2 p-value from likelihood ratio test.

### Sensitivity analysis

For Model 2, varying the age breakpoint to 5 and 7 years changed the *λ_1_* estimate between 15.5% and 15.1%, but the value of *λ_2_* was quite sensitive to changes in the age breakpoint, ranging from 10.4% to 6.7%.

### JE vaccination

Overall, 514 (64.5%) children had received JE vaccination. JE vaccination status was missing for 23 (2.9%) children. Among children aged 1 year, 35% of children had received JE vaccine, while above the age of 3 years, between 73% and 81% of children were vaccinated. Children vaccinated against JE were more likely to be seropositive (56.8% vs 41.9%, OR = 1.83, 95% CI: 1.34–2.50). However, this association was markedly reduced after adjusting for differences in the age distribution of vaccinees and non-vaccinees (age-adjusted OR = 1.22, 95% CI: 0.87–1.73).

**Figure 2:**
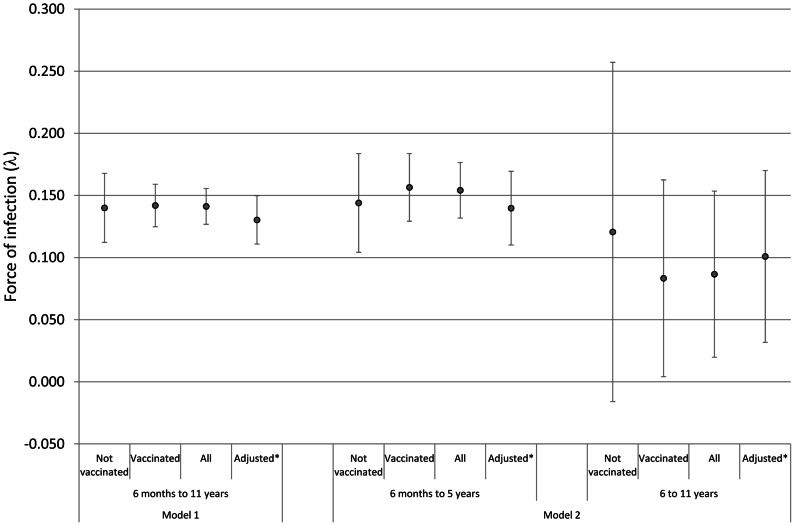
Force of dengue infection estimates and 95% CIs by JE vaccination status. Model 1: constant force of infection; Model 2: different forces of infection above and below 6 years. Figure 2 footnote: * Estimates from a model adjusting for JE vaccination status.

There was no evidence that JE vaccination history had any influence on dengue force of infection estimates (Figure 2). The force of infection parameters for JE vaccinees and non-vaccinees were similar for Model 1 assuming a constant force of infection. For Model 2, the force of infection was somewhat lower among JE vaccinees aged 6 to <12 years, but there was little statistical support for the interaction parameter (LR test p-value = 0.861). Adjusting for JE vaccination status had little influence on the force of infection estimates.

### Ratio of infections to notified cases

**Figure 3:**
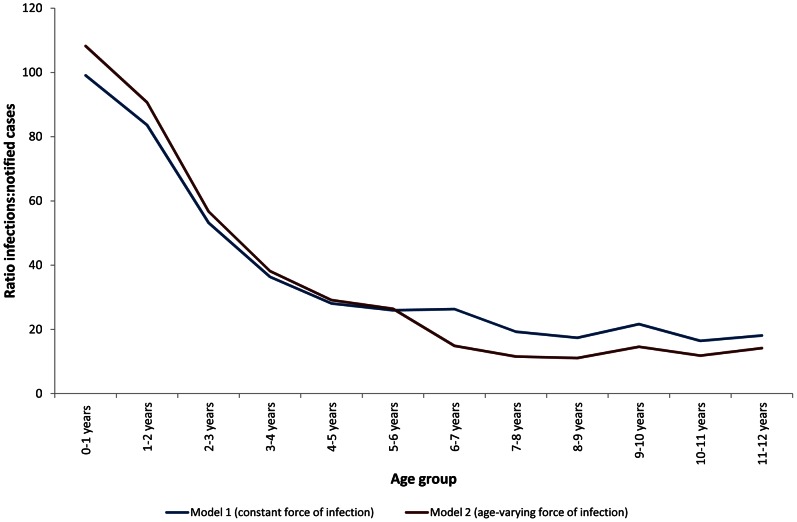
Ratio of dengue infections to notified cases by age in Colombo Municipal Council, 2009. Blue line: Model 1 (constant force of infection); Red line: Model 2 (different forces of infection above and below 6 years).

We estimated that approximately 27,000 primary dengue infections occurred in children aged six months to 11 years in CMC in 2009. The estimates were similar for Model 1 (27,913) and Model 2 (26,185), although the latter estimated a higher number of infections in children <6 years and fewer infections among those aged 6 years and above compared with Model 1. In the same year, 878 cases of DF/DHF were notified to the Ministry of Health. Thus, for every case of DF/DHF notified, an additional 30 primary dengue infections occur in the community. There was evidence that this ratio was age dependent, being much higher in the first two years of life than at older ages (Figure 3). Based on Model 1, we estimated an overall primary infection rate in children <12 years of 68 per 1000 children per year (95% CI: 65–70), as compared with a rate of 2.1 notified cases per 100,000 children per year. Primary infection rates based on Model 2 were similar (64 per 1000 children per year)

## Discussion

To our knowledge, this is the first study from the Asian subcontinent to present data on dengue seroconversion rates among children. Our study population also includes pre-school age children, which have not always been included in similar studies in other settings. Our findings indicate that transmission of dengue among the paediatric population of Colombo is high; among dengue naïve children, approximately 14% experience their first dengue infection (seroconvert) within 12 months. There was some suggestion in our data that this risk could be age-dependent; from our Model 2 estimates, the rate of seroconversion among children aged 6 to <12 years was lower at 8%. Although there was only weak statistical support for this age dependence, our sample size was modest and our study may have lacked power to detect age-dependent effects. Nevertheless, Model 2 predictions did provide a slightly better visual fit to the data at older ages, while the marked change in the force of infection parameters with age suggest that a lower rate of seroconversion among those aged 6 years and above could be plausible. If true, this would indicate a lower infection pressure at older ages. A possible reason for this could be changes in behaviour with age; school-age children spend more time outside the home, which may reduce exposure to bites by the primarily domestic, indoor-biting *Aedes aegypti* mosquito, the main dengue vector in Sri Lanka. Studies in other countries have indicated that dengue transmission clusters primarily around the home [9], [10], although house-to-house movements appear to be important for maintaining transmission in an urban environment [11]. Despite this, the two models provide similar estimates of the expected number of primary infections by age 12. A reduction in the rate of seroconversion above 6 years of age has a small impact, because most children experience their first infection at a younger age; the median age at first infection in this population was less than 5 years.

Our findings are comparable to those of other studies in Southeast Asia and Brazil. The median age at first infection is less than 5 years in Colombo. A recent seroprevalence survey in Recife, Brazil, indicated that over 50% of children in areas of intermediate and high deprivation are seropositive before the age of 10 years [12], while a recent study in Vietnam found that seroprevalence was approximately 50% by the time children enter primary school [13]. Seroprevalences of 23%, 9% and 17% at 9, 12 and 18 months of age were reported in a recent study in Bangkok, similar, though somewhat lower, than those found in our study [14]. Our force of infection estimates are also similar to those in other studies. Using similar methods with seroprevalence data, Braga et al. estimated the force of infection across all ages in a deprived area of Recife at 17%, with lower forces of infection in wealthier areas of the city [12]. Other studies have used longitudinal approaches to measure seroconversion rates empirically. Using repeat serological surveys two years apart, Teixeira et al. recently reported a seroconversion rate equivalent to 17% per year among children aged <3 years in Salvador, Brazil. In Vietnam, cohort studies have reported one-year seroconversion rates of 11% among children aged 2 to 15 years in the Mekong Delta [15], and 17% among primary school children aged 7 years and above in the southeastern coastal area. [13]. Among elementary schoolchildren in Kamphaeng Phet, Thailand, dengue infection incidence varied between 7.9% and 2.1% per year between 1998 and 2000, although this latter study did not report results separately for dengue-naïve and previously infected children [16].

We estimate that there are approximately 30 primary dengue infections in the community for every DF/DHF case notified to national surveillance. This ratio was much higher among children less than two years of age than in older age groups. This is partly because older children are more likely to experience secondary infections, which we were unable to estimate from our data. Our figures thus underestimate of the true ratio of infections to notifications at older ages However, the ratio was four times higher among children <2 years compared with those aged 6 years and above, which suggests a real differences even accounting for additional secondary infections at older ages. Such a difference is likely to result from a higher ratio of asymptomatic to clinical dengue among very young children; older age has been found to carry a greater risk of symptomatic dengue given infection, both in Latin America [17] and Southeast Asia [18]. The high burden of subclinical dengue, particularly at younger ages, represents a large pool of undetected infection. The importance of this pool of asymptomatic infection for transmission of dengue viruses remains to be determined.

Our study is subject to a number of limitations. Firstly, our serology results were from a flavivirus rather than dengue-specific ELISA. This could result in inaccurate force of infection estimates if antibodies to flaviviruses other than dengue were being detected. The most important of these is JE virus, which is endemic in Sri Lanka. If substantial levels of seropositivity against JE virus were being detected, we would expect that the force of infection estimates would differ between those with and without evidence of JE antibodies. We investigated this using JE vaccination history as a proxy for JE virus seropositivity and we found no evidence for such an effect, indicating that primarily dengue infections were being detected by our ELISA. In addition, high levels of JE virus circulation in our study are unlikely, as this virus is largely confined to rural areas of the country. An analogous finding was reported from a study in the Amazonian basin, in which dengue virus seropositivity was not affected by prior yellow fever vaccination [19]. In addition, we assumed that maternal antibody wanes by 6 months of age. The large drop in seroprevalence between 0 and 6 months suggests that this is a reasonable assumption. However, studies have shown persistence of maternal antibody in a minority of children beyond 12 months [14], [20], [21]. It is therefore possible that our models overestimate seroprevalence at these ages, which would have resulted in an underestimate of the rates of seroconversion.

Unlike in other childhood immunizing infections, a first infection with dengue does not provide long-term protection from re-infection, as individuals are still susceptible to infection with other dengue serotypes. Diagnosis of past dengue infection is complicated by the fact that, beyond the first infection, no method can reliably determine the serotypes to which an individual has been exposed. We were thus unable to estimate serotype-specific forces of infection; our estimates are better interpreted as the average force of first infection with any serotype. For this reason, we are also unable to comment on the risk of infection among seroconverters, which would require longitudinal approaches and/or diagnostics that are better able to differentiate between primary and secondary infections. Ferguson et al. previously estimated strain-specific forces of infection in Thai children under 10 years by applying a mathematical model to data from cross-sectional serological samples tested by plaque reduction neutralization tests, which can differentiate between strain-specific monotypic infections and multitypic infections [22]. Their serotype-specific force of infection estimates ranged from 0.01 to 0.1, and were highest for the DEN-2 serotype. Strain-specific forces of infection will depend on the relative frequency of circulation of each serotype, as well as any interactions between strains.

Our model assumes that the force of infection is constant over time. This may not be true in highly epidemic years, or if changes to the force of infection occur because of shifts in the predominant circulating strains. Such effects would likely result in cohort effects in the data, such as spikes in the prevalence of seropositivity in children of a specific age. Sri Lanka experienced a number of highly epidemic years prior to the study, most notably in 2004, but there is little evidence in our data of such cohort effects, suggesting that any temporal changes in the force of infection are minimal.

In estimating the number of dengue infections, we have extrapolated results from MC Ward 33 to the entire CMC region. It is possible that the force of infection in other areas of Colombo differs from that in our study area, because of differences in demographic or socioeconomic characteristics, or population and/or vector densities, although we have little reason to believe that our results would not be applicable to other areas of CMC.

Our study has important implications, both for understanding dengue transmission and developing control strategies. Our findings confirm that there is a high infection pressure at young ages, as more than half of children experience their first infection before the age of 5 years. This age group is also at highest risk of mortality. The high level of transmission at very young ages, suggests that control strategies and future vaccination schedules that focus these age groups are likely to provide the most benefit. It also suggests that transmission in Colombo primarily occurs in and around the home, as more than half of children seroconvert before they reach school age. Finally, a number of studies have indicated that transitions in dengue epidemiology, characterized by shifts in the average age of cases, are related to the force of infection [23] [24]. Brazil, where historically adults contribute the majority of cases, is currently seeing a trend towards infection and more severe disease at younger ages [24], [25]. Conversely, secular decreases in birth rates in Thailand are thought to be linked with declines in the force of infection and a resulting increase in the average age of dengue infection [23]. In Sri Lanka, high forces of infection, the predominance of cases in childhood, and a recent trend towards a greater contribution to the caseload among adults all indicate that the epidemic is at an equivalent stage to the well-established epidemics in Southeast Asia.

## Supporting Information

Checklist S1 STROBE checklist.
(DOC)
