# Supplementary material for: Estimates of Dengue Force of Infection in Children in Colombo, Sri Lanka
Source: PLoS Negl Trop Dis. 2013 Jun 6;7(6):e2259. doi: 10.1371/journal.pntd.0002259 (PMC3674987; doi:10.1371/journal.pntd.0002259)
Supplement: Checklist S1 — STROBE checklist. (DOC) [file pntd.0002259.s001.doc]

STROBE Statement—Checklist of items that should be included in reports of ***cross-sectional studies***

|  | Item No | Recommendation |
| --- | --- | --- |
| **Title and abstract** | 1 | (*a*) Indicate the study’s design with a commonly used term in the title or the abstract  *Although we use data from a cross-sectional serological survey, the purpose of the paper is to apply a model to estimate force of infection, rather than infer characteristics of the population. Our title reflects this more specific objective so we have not included the study design in the title.* |
|  |  | (*b*) Provide in the abstract an informative and balanced summary of what was done and what was found  *P2* |
| Introduction | | |
| Background/rationale | 2 | Explain the scientific background and rationale for the investigation being reported  *P3* |
| Objectives | 3 | State specific objectives, including any prespecified hypotheses  *P3 (introduction, last para), p4 (data analysis, first para), p6 (sensitivity analysis, second para)* |
| Methods | | |
| Study design | 4 | Present key elements of study design early in the paper  *P3-4 (methods, seroprevalence survey)* |
| Setting | 5 | Describe the setting, locations, and relevant dates, including periods of recruitment, exposure, follow-up, and data collection  *P3-4 (methods, seroprevalence survey)* |
| Participants | 6 | (*a*) Give the eligibility criteria, and the sources and methods of selection of participants  *P3-4 (methods, seroprevalence survey)* |
| Variables | 7 | Clearly define all outcomes, exposures, predictors, potential confounders, and effect modifiers. Give diagnostic criteria, if applicable  *P5 (methods, laboratory analysis, data analysis), p5 (methods, sensitivity analysis)* |
| Data sources/ measurement | 8* | For each variable of interest, give sources of data and details of methods of assessment (measurement). Describe comparability of assessment methods if there is more than one group  *P5 (methods, laboratory analysis, data analysis), p5 (methods, sensitivity analysis)* |
| Bias | 9 | Describe any efforts to address potential sources of bias  *P5 (methods, sensitivity analysis)* |
| Study size | 10 | Explain how the study size was arrived at  *The seroprevalence survey was done as part of a cohort study. The sample size was based on the main objective of the cohort study, which is beyond the scope of this paper. The protocol for the cohort study, sample size and selection procedures for participants are described in full in a separate manuscript being reviewed by a different journal. We can provide a draft of that manuscript for information if needed.* |
| Quantitative variables | 11 | Explain how quantitative variables were handled in the analyses. If applicable, describe which groupings were chosen and why  *P5 last para (methods, data analysis)* |
| Statistical methods | 12 | (*a*) Describe all statistical methods, including those used to control for confounding  *P5 (methods, data analysis), p5 (methods, sensitivity analysis)* |
|  |  | (*b*) Describe any methods used to examine subgroups and interactions  *P5 (methods, data analysis), p6 (methods, sensitivity analysis)* |
|  |  | (*c*) Explain how missing data were addressed  *N/A* |
|  |  | (*d*) If applicable, describe analytical methods taking account of sampling strategy  *P5(methods, data analysis, last para)* |
|  |  | (*e*) Describe any sensitivity analyses  *P5 (methods, sensitivity analysis)* |
| Results | | |
| Participants | 13* | (a) Report numbers of individuals at each stage of study—eg numbers potentially eligible, examined for eligibility, confirmed eligible, included in the study, completing follow-up, and analysed  *The seroprevalence survey was done as part of a cohort study. The protocol for the cohort study, sample size and selection procedures for participants are described in full in a separate manuscript being reviewed by a different journal. We can provide a draft of that manuscript for information if needed. We have not included this information here as it is not directly pertinent to the paper’s objectives* |
|  |  | (b) Give reasons for non-participation at each stage  *See (a) above* |
|  |  | (c) Consider use of a flow diagram  *See (a) above* |
| Descriptive data | 14* | (a) Give characteristics of study participants (eg demographic, clinical, social) and information on exposures and potential confounders  *See 13a above. The characteristics of the cohort, together with a comparison of the cohort and census populations in terms of demographic, socioeconomic and healthcare usage characteristics is given in a separate paper describing the study protocol* |
|  |  | (b) Indicate number of participants with missing data for each variable of interest  *P6 (results, JE vaccination)* |
| Outcome data | 15* | Report numbers of outcome events or summary measures  *P6 (results, first para)* |
| Main results | 16 | (*a*) Give unadjusted estimates and, if applicable, confounder-adjusted estimates and their precision (eg, 95% confidence interval). Make clear which confounders were adjusted for and why they were included  P6 (results, logistic model, sensitivity analysis, JE vaccination, table 2, figure 1 and figure 2) |
|  |  | (*b*) Report category boundaries when continuous variables were categorized |
|  |  | (*c*) If relevant, consider translating estimates of relative risk into absolute risk for a meaningful time period  *N/A* |
| Other analyses | 17 | Report other analyses done—eg analyses of subgroups and interactions, and sensitivity analyses  *P6 (results, sensitivity analysis, JE vaccination)* |
| Discussion | | |
| Key results | 18 | Summarise key results with reference to study objectives  *P7 (discussion, para 1)* |
| Limitations | 19 | Discuss limitations of the study, taking into account sources of potential bias or imprecision. Discuss both direction and magnitude of any potential bias |
| Interpretation | 20 | Give a cautious overall interpretation of results considering objectives, limitations, multiplicity of analyses, results from similar studies, and other relevant evidence  *P8 para 3, p9 para 1-3* |
| Generalisability | 21 | Discuss the generalisability (external validity) of the study results  *P8 para 1, p9 para 3* |
| Other information | | |
| Funding | 22 | Give the source of funding and the role of the funders for the present study and, if applicable, for the original study on which the present article is based  *See submission form* |

*Give information separately for exposed and unexposed groups.

**Note:** An Explanation and Elaboration article discusses each checklist item and gives methodological background and published examples of transparent reporting. The STROBE checklist is best used in conjunction with this article (freely available on the Web sites of PLoS Medicine at http://www.plosmedicine.org/, Annals of Internal Medicine at http://www.annals.org/, and Epidemiology at http://www.epidem.com/). Information on the STROBE Initiative is available at www.strobe-statement.org.
